# Comparison of confidence interval methods for an intra-class correlation coefficient (ICC)

**DOI:** 10.1186/1471-2288-14-121

**Published:** 2014-11-22

**Authors:** Alexei C Ionan, Mei-Yin C Polley, Lisa M McShane, Kevin K Dobbin

**Affiliations:** Department of Statistics, University of Georgia, Athens, GA USA; Biometric Research Branch, National Cancer Institute, Rockville, MD USA; Department of Epidemiology and Biostatistics, University of Georgia, Athens, GA USA

**Keywords:** Confidence interval, Credible interval, Generalized confidence interval, Intraclass correlation coefficient, Modified large sample

## Abstract

**Background:**

The intraclass correlation coefficient (ICC) is widely used in biomedical research to assess the reproducibility of measurements between raters, labs, technicians, or devices. For example, in an inter-rater reliability study, a high ICC value means that noise variability (between-raters and within-raters) is small relative to variability from patient to patient. A confidence interval or Bayesian credible interval for the ICC is a commonly reported summary. Such intervals can be constructed employing either frequentist or Bayesian methodologies.

**Methods:**

This study examines the performance of three different methods for constructing an interval in a two-way, crossed, random effects model without interaction: the Generalized Confidence Interval method (GCI), the Modified Large Sample method (MLS), and a Bayesian method based on a noninformative prior distribution (NIB). Guidance is provided on interval construction method selection based on study design, sample size, and normality of the data. We compare the coverage probabilities and widths of the different interval methods.

**Results:**

We show that, for the two-way, crossed, random effects model without interaction, care is needed in interval method selection because the interval estimates do not always have properties that the user expects. While different methods generally perform well when there are a large number of levels of each factor, large differences between the methods emerge when the number of one or more factors is limited. In addition, all methods are shown to lack robustness to certain hard-to-detect violations of normality when the sample size is limited.

**Conclusions:**

Decision rules and software programs for interval construction are provided for practical implementation in the two-way, crossed, random effects model without interaction. All interval methods perform similarly when the data are normal and there are sufficient numbers of levels of each factor. The MLS and GCI methods outperform the NIB when one of the factors has a limited number of levels and the data are normally distributed or nearly normally distributed. None of the methods work well if the number of levels of a factor are limited and data are markedly non-normal. The software programs are implemented in the popular R language.

**Electronic supplementary material:**

The online version of this article (doi:10.1186/1471-2288-14-121) contains supplementary material, which is available to authorized users.

## Background

Biological and physical quantities assessed for scientific studies must be measured with sufficient reproducibility for the study to produce meaningful results. For example, biological markers (“biomarkers”) are studied for many medical applications, including disease risk prediction, diagnosis, prognosis, monitoring, or optimal therapy selection. Variation in measurements occurs for numerous reasons. The measurements might have been made on different devices, may have involved subjective judgment of human raters (e.g., a pathologist assessing the number of tumor cells in a biopsy), or might have been made in different laboratories using different procedures. As another example, psychological instruments often score patients based on multi-item questionnaires completed by medical professionals. Variation in the resulting scores can be attributed to both variation among the patients and variation among the medical professionals performing the assessments. In many settings, it is not realistic to expect perfect concordance among replicate measurements, but one needs to achieve a level of reliability sufficient for the application area, such as a clinical setting. A common approach to quantify the reliability of a measurement process is to calculate the intraclass correlation coefficient (ICC) along with a confidence interval [1–4].

An interval can be constructed for the ICC using frequentist or Bayesian methods. Frequentist methods assure that the probability that the interval contains the parameter if the experiment is repeated many times is the nominal confidence level (e.g., 95%). In contrast to Frequentist methods, Bayesian methods provide a probability distribution for the parameter itself, given the data and the prior uncertainty. The distribution can be summarized by a credible interval, which reflects a nominal probability (e.g., 95%) region for the distribution. When little is known about the parameter of interest a priori, then a non-informative prior, which is often provided in the statistical software, can be used to construct the interval. The relative advantages of noninformative Bayesian and frequentist approaches in general are discussed in Berger [5] Chapter 4, Carlin and Louis [6] (Section 1.4), and elsewhere. General comparisons of the different approaches are beyond the scope of this paper. This paper focuses on two issues of applied interest discussed in the next paragraph.

Two critical and inter-related characteristics of a confidence interval method are (1) the coverage probability, and (2) the interval width. The coverage probability of a method should exactly match the confidence level, such as 95%. Coverage probability is a frequentist concept since the parameter is treated as a fixed number. The interval width is important to consider when comparing intervals because one often wants the shortest possible interval that maintains the nominal coverage. Coverage probability and interval width are important and relevant from both frequentist and objective Bayesian perspectives [7–13]. Frequentist coverage probabilities are interpretable in the Bayesian framework as well [14].

We study two applications in detail. The first application is a study by Barzman et al. [15]. They evaluated the Brief Rating of Aggression by Children and Adolescents (BRACHA), a 14-item questionnaire instrument scored by emergency room staffers. BRACHA scores can be influenced by both the child being assessed and the adult performing the assessment. Interest was in whether different adult staffers scored the children in a similar way, as summarized by the intraclass correlation coefficient. These data were originally analyzed using Bayesian credible interval methods. The second application is the National Cancer Institute’s Director’s Challenge reproducibility study [16]. In this study, tissue samples were subdivided into separate sections, sections distributed to four laboratories, and microarray analysis performed at each laboratory. Interest was in whether different laboratories produced similar gene expression measurements for individual patients.

This paper considers the setting of a two factor, crossed, random effects model without interaction. We focus on this setting because it arises frequently in practical applications of interest [15–17], and because this focus enables us to examine different aspects of study design, data distribution, and Bayesian priors, without the scope of the paper becoming unwieldy. For the purposes of this study, we assume this model is appropriate for the data; the process of selecting an appropriate statistical model and agreement measure are outside the scope of this paper and are discussed thoroughly elsewhere [18, 19]. A random effects model is appropriate when each factor represents a random sample from a larger population [20]; for example, a factor may represent labs randomly drawn from all labs that could perform the assay. If the population of labs is small, a finite population adjustment is possible [21], but rarely used in practice. If for some factors random sampling is not an appropriate assumption, then fixed-effects or mixed models can be used. Reproducibility methods for fixed and mixed models are discussed elsewhere [19, 22].

Confidence interval performance can be affected by both the study design used and the distribution of the data. If the study design has a limited number of levels of one or both factors, then this can impact interval performance. In practice, it is common that one factor will have a very small number of levels. The distribution of the data is assumed to be normally distributed and a violation of normality can impact coverage. Also, if one variance component is large or small relative to the others, resulting in different values of the ICC, then this can impact coverage as well. Different variance parameters and a range of model violations are studied using simulation and application. These studies lead to relatively simple and straightforward advice on which interval procedure will produce an interval with good performance characteristics. Also presented are cautionary notes about when examined methods will perform poorly.

The history of the development of the methods compared in this paper is briefly reviewed. The Modified Large Sample procedure for the two-way layout without interaction was developed in [23], and is based on earlier work of [24] using exact statistical methods. The Generalized Confidence Interval procedure for the two-way layout without interaction is presented in [25], and is based on a modification of a related method in [26], and the foundational work in [27]. Bayesian methods based on Markov Chain Monte Carlo are described in [28], were previously popularized in [29] and [30], and grow out of earlier work such as [31]. Bayesian intervals can be constructed with a variety of packages in R, such as MCMCglmm, or the popular software based on BUGS (Bayesian inference Using Gibbs Sampling), such as OpenBUGS [32], WinBUGS [33], or JAGS. The frequentist modified large sample (MLS) [24] and generalized confidence interval (GCI) [27] methods can be implemented using SAS version 9.3 VARCOMP procedure, or with the R programs provided with this manuscript.

This paper is organized as follows: Section 2 presents the model, briefly outlines the methods, and also presents the simulation settings. Section 3 presents the results of the Monte Carlo investigations. Section 4 presents real data applications. Section 5 presents discussion of the results. Section 6 presents conclusions. Mathematical details appear in the Additional file 1. Supplemental simulation details appear in Additional file 2.

## Methods

The model for the data is1

where μ is the overall mean,  are the effects of the patients (or biological samples, etc.),  are the effects of the laboratories (or raters or instruments, etc.), and  are within-laboratory (or within-rater, etc.) experimental errors. The standard random effects model assumptions are that  and  where all random variables are mutually independent. The between-laboratory intraclass correlation is  and the within-laboratory intraclass correlation is  The analysis of variance for the model is presented in Table 1.

**Table 1:** **Analysis of variance**

Source	DF^a^	Sum of squares	MS^b^	EMS^c^
Patient	*b*_0_ - 1			
Lab/rater	*l*_0_ - 1			
Error	*r*_0_ *b*_0_ *l*_0_ - *b*_0_ - *l*_0_ + 1			

^a^DF is degrees of freedom; ^b^MS is observed mean squares; ^c^EMS is expected means squares.

Notation: *y*_*b* • •_ is the average over *l* and *s* for fixed *b*.

The  is the variance between biological samples. For measurements to be reproducible, this variance must be large relative to the other sources of variability present. If  is close to zero, so that the population is homogeneous, then reproducibility will be poor. If  is larger, and the other sources of variability are controlled adequately, then good reproducibility is possible. Universal heuristics for defining good reproducibility in all cases are not available, but in some cases historical ICC values and/or clinical relevance may help guide appropriate ranges (e.g., [19]).

### Comparison measures

Coverage probabilities and average interval widths over a range of plausible true parameter values are compared. The coverage level is set to 95%. These are frequentist measures that answer the critical, concrete questions:

1. Will an interval constructed in this way have a 95% coverage probability, or will the coverage be lower or higher than 95%?
2. Will an interval constructed in this way be as narrow as possible, reflecting the strongest possible conclusions that can be drawn from the data?

The coverage probability of a statistical procedure for interval construction is defined as the probability that the constructed interval will contain the parameter. One final note along these lines; the summary statistics presented in Tables 2 and 3 below can be viewed as components of the Bayes risk relative to a true prior (versus the “working prior” used for estimation), a criterion recommended by Samaniego [14] for comparison of frequentist and Bayesian procedures (Additional file 1: Section S4).

**Table 2:** **Normal simulation table**

		b_0_= 48, l_0_= 3, r_0_= 1		b_0_= 96, l_0_= 6, r_0_= 1	
ICC_w_	Method	Coverage	Average width (SEM)	Coverage	Average width (SEM)
0.99	GPQ	0.949	0.755 (0.0014)	0.947	0.523 (0.0009)
0.99	MLS	0.950	0.758 (0.0014)	0.948	0.525 (0.0009)
0.99	Bayes	0.858	0.825 (0.0012)	0.930	0.570 (0.0009)
0.90	GPQ	0.943	0.685 (0.0014)	0.948	0.448 (0.0010)
0.90	MLS	0.946	0.690 (0.0014)	0.949	0.450 (0.0010)
0.90	Bayes	0.858	0.788 (0.0010)	0.943	0.497 (0.0010)
0.80	GPQ	0.955	0.595 (0.0014)	0.943	0.331 (0.0009)
0.80	MLS	0.957	0.602 (0.0014)	0.946	0.334 (0.0009)
0.80	Bayes	0.848	0.749 (0.0008)	0.956	0.378 (0.0010)
0.71	GPQ	0.959	0.373 (0.0011)	0.954	0.156 (0.0002)
0.71	MLS	0.968	0.377 (0.0012)	0.957	0.156 (0.0002)
0.71	Bayes	0.933	0.678 (0.0009)	0.964	0.169 (0.0003)

ICC_b_ = 0.70 setting. Highlighted are coverages below 90%. The means of the ICC_b_ point estimates when b_0_ = 48, l_0_ = 3 were 0.74, 0.72, 0.70 and 0.69, with standard deviations 0.17, 0.14, 0.09 and 0.06 as the values of the ICC_w_ decreased from 0.99 to 0.71. When b_0_ = 96, l_0_ = 6 the means of the ICC_b_ estimates were 0.72, 0.71, 0.70 and 0.70 with standard deviations 0.12, 0.10, 0.06, and 0.04 as the ICC_w_ decreased from 0.99 to 0.71.

**Table 3:** **Simulation study with uniform and gamma models**

		Uniform model				Gamma model			
		b_0_= 48, l_0_= 3, r_0_= 1		b_0_= 96, l_0_= 6, r_0_= 1		Low skew		High skew	
*ICC*_*w*_	Method	Cov.	Wid.	Cov.	Wid.	Cov.	Wid.	Cov.	Wid.
0.99	GPQ	0.976	0.768	0.986	0.544	0.938	0.749	0.918	0.731
	MLS	0.977	0.771	0.986	0.546	0.941	0.752	0.922	0.734
	Bayes	0.873	0.823	0.985	0.593	0.856	0.825	0.849	0.823
0.90	GPQ	0.977	0.705	0.985	0.464	0.935	0.684	0.919	0.670
	MLS	0.979	0.710	0.986	0.467	0.937	0.689	0.926	0.675
	Bayes	0.879	0.790	0.986	0.516	0.849	0.788	0.843	0.767
0.80	GPQ	0.980	0.614	0.990	0.344	0.931	0.600	0.901	0.586
	MLS	0.981	0.621	0.990	0.347	0.936	0.607	0.908	0.593
	Bayes	0.883	0.753	0.992	0.394	0.932	0.752	0.805	0.745
0.71	GPQ	0.987	0.379	0.994	0.156	0.903	0.422	0.866	0.421
	MLS	0.992	0.384	0.996	0.156	0.918	0.429	0.884	0.428
	Bayes	0.958	0.686	0.996	0.169	0.859	0.699	0.832	0.695

Comparison of MLS, GPQ and Bayes method performance on uniform and gamma data. Nominal 95% confidence intervals for ICC_b_. Coverages and average widths calculated from 10,000 simulations. In each case, ICC_b_ = 0.70. Study designs have 48 biological replicates and 3 labs for a total of 144 observations, and 96 biological replicates and 6 labs for a total of 576 observations. Means and standard deviations of the point estimates of the *ICC*_*b*_ for each setting are presented in a Additional file 2: Excel file.

### Frequentist interval methods

A generalized confidence interval (GCI) is an extension of the traditional concept of a confidence interval. Traditional confidence intervals can be constructed when there is a pivotal quantity with a known distribution free of nuisance parameters. There is no such pivot for *ICC*_*b*_. The GCI method is based on a generalized pivotal quantity *G*[25, 27], which is a generalization of the usual pivot [34]. Define *F*_*G*_ as the cumulative distribution function for *G*. The formula for *G* is shown in Appendix A; the distribution of *G* is a function of chi-squared random variables. Monte Carlo methods can be used to estimate quantiles of *G*, say  for the *p*th quantile. The equal-tailed (1 - *α*)100% GCI is then,

The modified large sample (MLS) method is an extension of traditional confidence interval methods, which do not work well for the *ICC*_*b*_. The MLS approach is to construct the traditional asymptotic limits for the *ICC*_*b*_, and then modify these limits to improve the small-sample performance of the intervals. In particular, the limits are modified so that when all but one of the variance parameters is zero, the interval is exact [24]. The specific approach for the *ICC*_*b*_ is given in Cappelleri and Ting [23]. The general form of the MLS interval is a function of the observed mean squares, and can be written:

where L and U are functions mapping 3-dimensional space to one-dimensional space, and ,  and  are mean squares defined in Table 1. Unlike the GCI approach, the MLS interval is constructed from closed formulae, which appear in Appendix B. The computational cost of constructing an interval using the MLS procedure is smaller than the GCI procedure in general.

### Bayesian interval methods

In contrast to the frequentist methods described above, the Bayesian methods available in MCMCglmm, WinBUGS, and similar software, are general and not specifically developed for the *ICC*_*b*_ application. They can be used to construct confidence intervals for variance components, or functions of variance components. The user must specify a prior distribution for the variance parameters, denoted  Then, given the data *D*, a posterior distribution for the variance parameters is calculated, namely

An explicit density formula will not generally exist. But Markov Chain Monte Carlo methods (e.g., Tierney [30]) can be used to generate a very large sample from this posterior distribution. Then, this sample can be used directly to estimate the posterior distribution of *ρ*_*b*_ = *ICC*_*b*_, that is, the density *f*(*ρ*_*b*_|*D*). The 95% credible interval will contain area 0.95 under the posterior density curve. The highest posterior density (HPD) credible intervals will be the shortest possible credible interval [5](p. 140).

Bayesian software have a variety of noninformative priors from which to choose. As discussed in the Additional file 1, we performed an extensive investigation of all the noninformative priors on variance components that were offered, using as guidance advice in [6] and [35]. In the Results presented in the paper, only the best performing noninformative prior is shown. This turned out to be a uniform prior on the standard deviations, that is, the improper prior:

The same prior was recommended by Gelman [35] (Section 7.1) for obtaining point estimates of individual variance parameters in a one-way analysis of variance, although in that context he warns that this prior can result in miscalibration if the number of groups is small. In particular, the estimate of a variance component for a factor with a small number of levels will tend to be high.

### Software

In this paper we developed our own programs for frequentist inference and these programs are available online at http://dobbinuga.com. The software SAS can also be used to construct MLS and GPQ intervals. Bayesian programs for constructing credible sets based on HPD regions include MCMCglmm [36] and winBUGS [33], among others. We use the MCMCglmm package to construct Bayesian HPD credible intervals. Implementation details are provided in the text. The simulation programs we wrote are available from the authors upon request.

### Simulation settings

In order to evaluate the different intervals, we looked at the performance metrics discussed above under the model assumption of Equation (1), and under violations of the model assumption. Simulations were run under the settings in Table 4. Parameter values used are discussed in Appendix C.

**Table 4:** **Simulation settings**

Model name	***B***_***b***_distribution	***L***_***l***_distribution	***e***_***blr***_distribution
Normal	Normal	Normal	Normal
Uniform	Uniform	Uniform	Uniform
Mixture normal	Mixture normal	Mixture normal	Mixture normal
Gamma low-skew	Gamma	Gamma	Gamma
Gamma high-skew	Gamma	Gamma	Gamma

For each row of the table, we examined *b*_0_ ∈ {48, 96}, *l*_0_ ∈ {3, 6}, *r*_0_ = 1 and *ICC*_*b*_ ∈ {0.70, 0.90}.

The value of *ICC*_*b*_ was examined at 0.7 and 0.9. These represent reproducibility levels typically encountered in practice. When *ICC*_*b*_ is 0.7, then the within-laboratory (or within-rater, etc.) *ICC*_*w*_ must be at least 0.70; we examined *ICC*_*w*_ at 0.71, 0.80, 0.90, and 0.99, representing a wide range of possible values. When *ICC*_*b*_ = 0.9, we examined *ICC*_*w*_ = 0.94.

The designs we examined had *b*_0_ as 48 or 96, representing moderate sized studies typically feasible for settings where resources are limited. The number of laboratories (or raters, etc.) used was 3 or 6, representing a setting where this number is restricted by logistics or costs.

## Results

### Under normality

We first examine the different confidence interval methods when the effects and errors are normally distributed, so that the model assumptions are correct. Table 2 shows the results when there are 48 samples and 3 laboratories. The *ICC*_*b*_ = 0.70. Similar results were found for *ICC*_*b*_ = 0.90 (Additional file 3). The coverage probabilities should be 95%. The GPQ method coverages are all within 0.01 of this target. All but one of the MLS coverages are within 0.01 of the target, with one setting being slightly conservative (coverage 0.968 when *ICC*_*w*_ = 0.71). The coverage probabilities of the Bayes intervals are below 95% in all cases, and below 90% in three of the four settings. The average width of each interval type decreases as the *ICC*_*w*_ decreases. In all 4 settings, the widths of the MLS and GPQ intervals are practically identical. But in each setting the Bayes width is wider. This is surprising since wider intervals usually correspond to higher coverage. The excess width of the Bayesian intervals increases as the *ICC*_*w*_ decreases, going from 0.825-0.758 = 0.067 (Bayes width minus MLS width) up to 0.678-0.377 = 0.301 as *ICC*_*w*_ goes from 0.99 down to 0.71.

Table 2 also shows the results when the number of laboratories is doubled to 6 and the number of samples increases to 96. The coverage probabilities of the GPQ and MLS methods are within 0.01 of the 95% nominal level in all cases. The Bayes methods are within 0.01 of the target in two of the four settings; in the other two settings, the Bayesian interval coverage is anticonservative when *ICC*_*w*_ = 0.99, and conservative when *ICC*_*w*_ = 0.71 (coverages 0.930 and 0.964, respectively). The Bayesian method performance improves with the larger sample size and number of labs. In terms of interval widths, the GPQ and MLS methods are again indistinguishable from one another. The Bayesian intervals are wider than the frequentist intervals in all scenarios.

### Under violations of normality

We consider performance under model violations, that is, when neither the effects nor the errors are distributed according to the assumed normal distribution.

We first consider the uniform distribution. Table 3 shows the results with 48 biological replicates and 3 laboratories. The GPQ and MLS methods both tend to have higher than nominal coverage, ranging from 0.976 to 0.992. The Bayesian method coverage is below 0.90 in three of the four settings, and is within 0.01 of the nominal in the other setting. The Bayesian methods only show minor improvement in coverage between the normal case and the uniform distribution case. As for interval width, the GPQ and MLS widths are again practically identical to one another throughout. The Bayesian widths are consistently larger. As was the case with the normal distribution setting, the Bayesian widths tend to both be wider and have lower coverage than the frequentist intervals.

When the number of biological replicates increases to 96 and the number of laboratories increases to 6 in the uniform model setting, the coverage probability for all methods increases (Table 3). In all cases, the coverage probability exceeds the nominal 95% level. The widths of the intervals are similar to those in Table 2 under the corresponding normal model. The Bayesian intervals are consistently wider than the frequentist intervals.

Table 3 also shows the results of comparison under the gamma model. The gamma distribution is intuitively a more serious violation of normality than the uniform distribution. When α = 3, the skewness is 1.15 (normal = 0) and the kurtosis is 5 (normal = 3). This is called the “high skew” model in the table (rightmost two columns). For the high skew setting, all methods have coverage probability below 93% across all scenarios. When *ICC*_*w*_ = 0.71, all methods have coverage below 90%. When the skewness and kurtosis are reduced (Table 3, “low skew” model with α =10 corresponding to skewness is 0.63 (normal = 0) the kurtosis is 3.6 (normal = 3)), the performance of all methods improve. Of note, the coverage probability of the frequentist methods are still below 92% when *ICC*_*w*_ = 0.71. The Bayesian method has lower coverage than the frequentist methods except for one case. Comparing the interval widths, the Bayesian methods consistently have wider intervals than the frequentist methods across all of these settings. The two frequentist methods have very similar mean widths. Overall, while the frequentist methods appear slightly preferable to the Bayesian methods, none is ideal in the presence of skewed data.

Importantly, note that the departure from normality in the high skew gamma data is hard to detect in an actual fitted dataset. For example, we generated 10,000 datasets from the high skew gamma model. We fit the model to each dataset and performed the Shapiro-Wilk’s normality test for the residuals. The mean p-value was 0.19, and the median was 0.08, and 55% of the p-values were above 0.05. For the low skew model, we did the same type of simulation and the mean Shapiro-Wilk’s residual p-value was 0.30 with a median of 0.20, and 69% of the p-values above 0.05.

A mixture normal distribution appeared similar to the normal distribution (Additional file 3).

### Real data application: Barzman et al. study

This study involved 24 children (on video) rated by 10 different emergency room staff members. First, we followed the analysis described in Barzman et al. [15]. The analysis of variance table is shown in Table 5. If we represent the variance between the children by  the variance between the staff members rating the videos by  and the error variance by  then the *ICC*_*b*_ is  The estimated *ICC*_*b*_ reported in the paper is 0.9099. The 95% credible interval using noninformative priors reported in the Barzman et al. [15] paper is (0.8416, 0.9526). The 95% GCI that we computed with our program in this case is (0.8510, 0.9569). The Bayesian interval is about 5% wider than the GCI in this case, which is a trivial difference. The Bayesian interval is shifted to the left, relative to the frequentist interval, corresponding to lower estimates of the ICC_b_. But the shift is very minor, and 96% of the GCI interval overlaps with the Bayesian interval, so that only 4% of the GCI interval does not overlap with the Bayesian interval.

**Table 5:** **ANOVA table from the Barzman et al.**[15] **study**

Source	DF	SS	MS
Raters	9	39.73	4.414
Children	23	2,195.35	95.450
Error	207	162.69	0.786

Since we have discovered that the ICC intervals can be sensitive to violations of normality, we analyzed the data to assess normality of the effects and errors. First, we analyzed transformations of the response variable using both the method of Box and Cox [37] and the modulus method [38]. Both methods indicated that the BRACHA scores *y* should be transformed to approximately *z* = (*y* + 0.5)^0.55^. Supporting the need for transformation, a test for regression curvature had p-value 0.004, a Shapiro-Wilk test on the residuals had p-value 0.001, and a non-constant variance score test had p-value 0.001. On looking back at the raw data, it was observed that one child had one extreme outlying score. The child’s scores were (0,0,0,0,0,0,0,0,0.5,3.5). The one 3.5 is an extreme which had the largest Cook’s distance (0.11). Hence, a single rater’s unusual observation may be driving the apparent normality violation. To keep the model balanced, we therefore deleted this child’s data (child 11), resulting in 23 children. Re-analyzing the data from scratch resulted in the same transformation of the BRACHA scores. However, the regression test for curvature had p-value of 0.43, the Shapiro-Wilk normality test on the residuals had p-value 0.51, and the non-constant variance score test had p-value 0.19. Thus, there is no longer any evidence of lack of normality. The mean squares were 8.3721, 0.3667, 0.0671 for the reduced dataset. The resulting 95% generalized confidence interval for *ICC*_*b*_ is (0.8423, 0.9542). Although it did not have a large impact on the confidence interval in this case, the process outlined here of carefully assessing normality and revising the analysis as needed, should be part of interval construction. The reason for the minor impact on the interval in this case, compared to the simulations, may be the large number of raters (10 raters).

### Real data application: NCI DC reproducibility study

The National Cancer Institute’s Director’s Challenge reproducibility study examined the reproducibility of 22,283 features represented on the Affymetrix U133A Genechip across a collection of frozen patient tissue samples. Unlike other technologies that measure the level of a single gene at a time, microarrays measure the levels of expressions of thousands of human genes simultaneously. The expression measurements are continuous, so that for each individual gene one can assess the reproducibility of the measurements for that particular gene across the different samples by calculating the *ICC*_*b*_. The result is 22,283 different reproducibility estimates, one for each feature. The NCI DC reproducibility study was one of the largest studies of the reproducibility of microarrays, and thus is of interest in terms of the strength of the conclusions we can draw. To this end, we constructed confidence intervals for all 22,283 features using both the frequentist and Bayesian approaches. For the confidence interval constructions, some samples were omitted to force the design to balance. The result was 4 labs and 11 samples for a total of 44 observations for each feature. Data were normalized as in Dobbin et al. [16] except that dChip [39] was used instead of MAS 5.0 (http://www.affymetrix.com/support/technical/whitepapers.affx). We first applied both Bayesian and frequentist methods to construct confidence intervals for each feature. Results are shown in Table 6. For features with reasonably high reproducibility (*ICC*_*b*_ > 0.52, top 2 quartiles of features) the interval widths for the GCI’s had lower mean and median than the corresponding Bayesian interval widths.

**Table 6:** **DC lung study results**

ICC_b_range	Frequency of features	Interval method	Mean pseudo-coverage	Mean width	Median width	SD widths
0.72-1	5,571	GCI	96.5%	0.439	0.446	0.131
		NIB	96.7%	0.520	0.530	0.154
0.52- < 0.72	5,571	GCI	97.2%	0.594	0.607	0.061
		NIB	94.8%	0.664	0.675	0.078
0.23- < 0.52	5,571	GCI	98.2%	0.594	0.616	0.068
		NIB	96.3%	0.591	0.619	0.109
0- < 0.23	5,571	GCI	99.2%	0.405	0.422	0.142
		NIB	85.9%	0.346	0.340	0.128

Summary of 22,283 confidence intervals, one for each feature, broken down by ICC_b_ quartiles. GCI is generalized confidence interval method, and NIB is Bayesian method with noninformative prior distribution. Pseudo-coverage is the proportion of times the full data ICC_b_ was contained in the interval.

In order to estimate the coverage probabilities of the DC reproducibility study intervals, we considered the 44 samples examined as a random sample from a finite population which consisted of all 69 tumor microarrays in the original dataset. For this “population” of 69 samples, the true *ICC*_*b*_ values were calculated from the unbalanced data, using the expected mean squares presented in [16]; we can call these values pseudo-parameters, to distinguish them from the true population parameters, which are unknown. The proportion of times the pseudo-parameters were contained in each interval was calculated; we term this pseudo-coverage. Note that pseudo-coverage is equal to the true coverage for the finite population of 69 samples. As shown in Table 6, for features with *ICC*_*b*_ > 0.72, representing the quartile with the highest reproducibility (highest pseudo-parameter values), the pseudo-coverage of the frequentist and Bayesian methods are similar (96.5% and 96.7%, respectively), but the GCI interval width mean is much smaller than the NIB interval width mean (0.439 versus 0.520, or 16% narrower GCI). These width differences are similar to those observed in the simulations. Interestingly, Table 6 also reveals that the NIB coverage breaks down (with coverage only 85.9%) when *ICC*_*b*_ ≤ 0.23, while the GCI maintains high coverage (with coverage 99.2%) in this setting. This observation suggests that the Bayesian methods may undercover when the point estimate of the *ICC*_*b*_ is small.

Because of the importance of normality of the data, we re-evaluated the DC reproducibility study more closely with this in mind. First we performed the method of Box and Cox [37] for the linear model of Equation 1 for each gene to assess the optimal normalizing transformation. The distribution of the Box-Cox lambda values is shown in the Additional file 1. There is some variation in the estimated optimal lambda values. They are centered near zero. Zero corresponds to the log transformation used in the previous analysis for all features. However, since normality is so important for ICC interval validity, we re-analyzed these data using the gene-specific Box-Cox transformations. We ran the Tukey interaction tests on all features, and all had p-values over 0.05, indicating no evidence of interaction effects. The resulting Shapiro Wilk test p-value distribution had mean of 0.44, and approximately 12% of features had a p-value below 0.05. There appeared to be no patterns in the Shapiro Wilk p-values that would be useful in identifying the normally distributed genes. Our conclusion is that the confidence intervals for most features should be valid, but that individual feature CI’s should be interpreted in the context of the corresponding Shapiro Wilk test p-value.

## Discussion

Two questions arise from these observations. (1) Why are the noninformative Bayesian methods performing poorly relative to the frequentist methods in some cases? (2) Why are both methods not robust to skewness and kurtosis?

For question 1, these results naturally led us to further investigate the Bayesian credible interval methodologies. When the Bayesian and frequentist intervals differed, the midpoints of the Bayesian intervals tended to be further from the true *ICC*_*b*_ than the midpoints of the frequentist intervals. The result we saw was wider intervals with poorer coverage. But why did this happen? Detailed discussion appears in the Additional file 1. In summary, we discovered potential reasons for the poor performance of the noninformative Bayesian priors. One issue is that *noninformative priors on variance components do not imply noninformative distributions on the ICC*. In fact, we derive these distributions in the Additional file 1 and show that they can be nearly point masses at 0 and 1. The one distribution where this is not the case is the one that works best in practice, namely, the uniform distribution on the standard deviation. But even this prior distribution on the *ICC*_*b*_ has most of its mass towards the edges of the unit interval (Additional file 1: Figure S2). That being said, this fact probably does not entirely explain the poor performance. The second potential issue is that *the Bayesian methods are not based on an underlying exact interval construction method, like the GCI and the MLS methods*. Put another way, the modified large sample method uses a “modified” version of the usual large sample method, whereas the Bayesian methods use an “unmodified” Bayesian computation. Indeed, since the GCI method is closely related to the nonparametric Bayesian method [34], it may be that nonparametric Bayesian methods can be used to adjust the Bayesian parametric intervals.

The lack of robustness to skewness and kurtosis may appear surprising given that analysis of variance in general is robust to these. However, since we are constructing a confidence interval for a ratio of variance components, this means that estimation becomes more unstable. For example, the MLS interval equation involves fourth order moments. In general, the higher order a moment is, the more difficult it is to estimate. The GCI method, while not relying explicitly on fourth order moments, relies on the assumption that the second order moments are chi-squared in order to estimate quantiles of the generalized pivot, which is conceptually quite similar to calculating a fourth order moment.

## Conclusions

**Figure 1:**
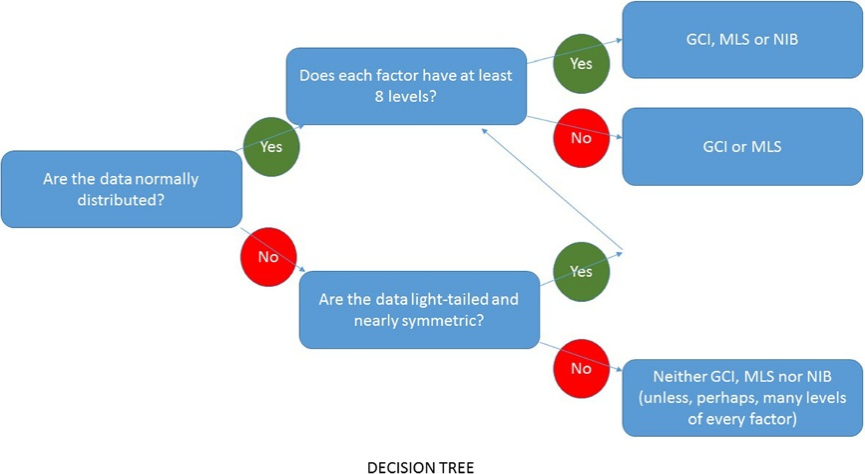
**Decision tree for confidence intervals for the ICC.** *Note that 8 is an estimate based on the simulations of this paper but may not be appropriate in all applications.

In this paper several methods for constructing intervals for the intraclass correlation coefficient were examined. Coverage probabilities and confidence interval widths were reported for the commonly encountered two-way, crossed-effects linear model without interaction. The Modified Large Sample (MLS), Generalized Confidence Interval (GCI) and noninformative Bayesian interval methods were evaluated. When model assumptions are true, we showed that the MLS and GCI methods perform well under a wide range of settings. Bayesian software with noninformative priors on variance components did not perform as well in most settings, often failing to achieve desired coverage and at the same time, counterintuitively, also resulting in wider average interval widths. Under model violations, it was shown that the methods performed similarly when there was small skewness and kurtosis. However, neither the frequentist nor the Bayesian methods were robust to hard-to-detect skewness and kurtosis when the number of levels of one factor is small. The methods were applied to two previously published reproducibility studies and new insights were gained. Future directions to improve the Bayesian approaches were suggested. A decision tree summarizing this paper’s findings is presented in Figure 1.

A number of commonly used noninformative Bayesian priors for variance components were studied. Results are in the Additional file 1. Bayesian priors for the intraclass correlation coefficient were derived mathematically for the inverse gamma and uniform (on standard deviations) priors. The commonly used inverse gamma prior on individual variance components resulted in an ICC prior very close to the extreme prior of two point masses: one at 0 and the other at 1. The inverse gamma (IG) prior was also found to lack scale invariance. Specifically, simply rescaling the data can drastically change the resulting (IG prior-based) interval for intraclass correlation coefficient (either ICC_b_ or ICC_w_). Moreover, this change is a function of the user-defined choice of non-informative prior parameter, so that IG(0.001,0.001) produces very different intervals than IG(0.01,0.01); a similar result was reported by Gelman [35] in the context of making inference about individual variance components. The uniform prior used in this paper does not result in a nearly degenerate prior for the ICC, is not affected by scale changes in the data, and is not sensitive to user-defined parameter choices (trivially, since there are none).

A question outside the scope of this paper is whether it is possible to develop Bayesian methods that would have performance comparable to the frequentist methods across all scenarios in terms of mean interval width and coverage probability. It is possible that at some point in the future such a method will be developed. One possibility mentioned in the discussion is adapting nonparametric Bayesian methods for random effects models to this setting (for discussion, see [40]). Another possibility, following a suggestion in Gelman [35], is to have a relatively minor modification of the prior and place uniform distributions on individual variance components with finite support, so that *σ* ~ *Uniform*(0, *k*) for some *k* > 0; a different constant *k* could be used for each variance component, and these would need to be chosen based on prior knowledge or on the data (e.g., empirical Bayes). Indeed, the utility of Bayesian methods in medical contexts where prior or expert knowledge is available is widely recognized. Further research in this direction seems needed.

In modeling laboratory reproducibility, we have assumed that the effect of a laboratory is represented by a tendency to score higher or lower than other laboratories across biological samples assayed. But laboratory effects may be represented in other ways. For example, it may be that some laboratories have higher variance in their measurements, but no systematic difference across individuals. Such a setting could be represented by a variance components model that allowed each lab to have its own within-laboratory measurement error variance (that is, in Equation (1), permit  to vary by laboratory). This would represent that lab’s ability to obtain replicable measurements in repeated assays. The null hypothesis that all within-lab variances are equal could be tested against the general alternative. Alternatively, the CCC could be used [18], as suggested by a referee. As another example, an interaction between labs and samples could be introduced into Equation 1 to represent lab-to-lab variation in ability to reproducibly measure individual samples, and indeed we have used a Tukey test to assess such interaction in the first application.

We used simulation to investigate whether we could develop post-hoc rules which could be used to select an interval construction method. Unlike Figure 1, these rules would be based on the values of the observed mean squares, in addition to the study design and normality assumptions. We were unable to come up with helpful rules that could be used in practice. But these results (not presented) suggested that the Bayesian methods tend to underperform more often when the laboratory variance estimate is large relative to the biological variance, and that the frequentist tend to underperform when the estimated biological variance is very large relative to the estimated laboratory variance. But we discourage investigators from using these broad observations in selecting a methodology, and recommend instead Figure 1.

The number 8 in Figure 1 as the cutoff number for how many levels are enough for the noninformative Bayesian method performance to match the frequentist is a best guess, and not a hard number based on theoretical results. However, we did run extended simulations with 4-16 laboratories, and these results are presented in the Additional files 4 and 5. With 8 levels (labs) it seems that one could safely conclude that the noninformative Bayesian, MLS and GCI would all be very similar and adequate under the normal model assumptions. The Bayesian coverages are similar to the frequentist for even 4-6 levels, but the Bayesian interval widths are noticeably wider.

## Appendix A: formula for the generalized pivotal quantity



where *c*_2_ = (*b*_0_ - 1)/(*l*_0_*r*_0_), *c*_3_ = (*b*_0_*l*_0_*r*_0_ - *b*_0_ - *l*_0_ + 1)/(*l*_0_*r*_0_), *c*_1_ = (*l*_0_ - 1)/(*b*_0_*r*_0_) and *c*_4_ = *c*_3_(*b*_0_*l*_0_*r*_0_ - *b*_0_ - *l*_0_ + 1)/*b*_0_. Here  and  are mutually independent given the observed mean squares. Generating a large number of (*W*_1_, *W*_2_, *W*_3_) triples (such as 100,000) by Monte Carlo, the generalized confidence interval is formed from the quantile function 

## Appendix B: modified large sample formula

The formula for the interval (L,U) is

where the constants *G*_2_, *F*_5_, *F*_4_, *H*_2_, *F*_6_, *F*_3_ are quantiles of F distributions as defined in the Additional file 1 and [25].

## Appendix C: simulation parameter settings

Additional file 1: Table S1 shows the complete list of simulation settings used. Simulation results not presented in the paper appear in the Additional file 3. For the simulations involving the normal distribution, data were generated as given in Equation 1 above.

The robustness of intervals to violations of the normality assumption was evaluated by generating effects and errors from uniform, mixture normal, and gamma distributions. Parameters settings were calculated to make the variances of the simulated biological effects, lab effects and measurement error exactly the same as in the normal simulations.

For a random variable X with the uniform distribution on the interval [-A,A], the variance is *Var*(*X*) = *A*^2^/3. This leads to the formulas

If the distribution of each effect in Equation (1) is uniform, instead of normal, then the marginal distribution of the responses, Y_blr_, are sums of uniform random variables. The marginal density is derived in Additional file 1: Section S5 and plotted in the Additional file 1.

A random variable X with a normal mixture distribution with means ± μ and standard deviations μ/3, and weights 0.5, will be bimodal. We can write the mixture normal as a hierarchical model with c ~ Bernoulli(0.5), and

Then E[*X*] = 0 and Var(*X*) = μ^2^*(10/9).

The resulting equations are  The marginal densities for Y_blr_ are also mixture normal (see Additional file 1: Section S5), and are shown in the Additional file 1.

Define Gamma(α,β) by the density function  Effect sizes and errors are generated by the following steps:

Note that *w* can be viewed as a mean-shifted version of  and since central moments are translation-invariant, the central moments of *w* are the same as the central moments of a Gamma(α,σ/ α^1/2^). As a result, E[*w*] = 0, Var(*w*) = σ^2^, skewness(*w*) =  and kurtosis(*w*) = 3 + 6/α [41] (p. 31). We keep β = 1. We let α = 1, 3, 10, 40. The marginal densities for *Y*_*blr*_ are discussed in Additional file 1: Section S5 and shown in the Additional file 1.

## Electronic supplementary material

Additional file 1:**Supplement includes additional discussion, simulations, data analysis details, figures and tables.**(PDF 855 KB)

Additional file 2:**Supplement presents the mean and standard deviation of the point estimates of the ICCb for different models and designs presented in the main paper.**(XLS 22 KB)

Additional file 3:**Supplement presents complete tables of the core simulations simulations from which the tables in the paper are a subset.**(XLS 62 KB)

Additional file 4:**Supplement is a table of results for designs with 8, 10, 12, 14 and 16 factor levels for laboratories.**(XLS 31 KB)

Additional file 5:**Supplement is a table of results for designs with 4, 5, 6 and 7 factor levels for laboratories.**(XLS 26 KB)
