# Supplementary material for: Comparison of confidence interval methods for an intra-class correlation coefficient (ICC)
Source: BMC Med Res Methodol. 2014 Nov 22;14:121. doi: 10.1186/1471-2288-14-121 (PMC4258044; doi:10.1186/1471-2288-14-121)
Supplement: Supplementary file 1 — Additional file 1:Supplement includes additional discussion, simulations, data analysis details, figures and tables.(PDF 855 KB) [file 12874_2014_1133_MOESM1_ESM.pdf]

Supplement to: Guidelines for constructing a confidence interval for the intra-class correlation coefficient (ICC)

Authors: Alexei C. Ionan, Mei-Yin C. Polley, Lisa M. McShane, Kevin K. Dobbin

Section	Page
S1: A Review of Frequentist Methods	1
S2: A Review of Bayesian Methods	3
S3: Investigation of the Bayesian Interval Methods	4
S4: Samaniego Criteria	11
S5: Description of Excel File	12
S6: Marginal distributions for simulation settings	13
S7: Table of simulation settings	13
S8: Supplementary Figures	14
S9: Data analysis details	15

Section S1: A REVIEW OF FREQUENTIST METHODS

There is no general formula for the exact distribution of the ICC which could be used for interval construction. The asymptotic distribution of the ICC (or a variance stabilized transformation of the ICC) can be derived from published results (e.g., Searle et al., 2006). But simplistic intervals constructed from these asymptotics can be substantially anticonservative (e.g., Zou and McDermott, 1999) ; this means, for example, that the probability that a nominal 95% confidence interval for the ICC contains the true ICC can be well below 95%. The rate of convergence of the ICC to these asymptotics is too slow for most practical situations. Two frequentist CI methods to address this problem are discussed in the next two paragraphs.

Uniform minimum variance unbiased (UMVU) estimators of each variance component in the model have been derived (e.g., Lehmann and Casella, 1998). For linear combinations of variance components, Graybill and Wang (1980) showed that exact confidence intervals can be built from linear combinations of UMVU estimates in special cases where some of the mean squares are exactly zero; specifically, when some mean squares are zero, then coefficients on the other mean squares that provide the exact coverage can be derived mathematically. Cycling through and setting different variance components to zero results in coefficient estimates for all mean squares. Putting these together results in a modification of the usual interval limits based on standard asymptotics. This approach is called the modified large sample (MLS) method. An MLS procedure for the ICC in the random two-way layout without interaction was developed in Cappelleri and Ting (2003).

Pivotal quantities are often used to construct confidence intervals. A pivotal quantity is a function of the parameter and the data with a known distribution (e.g., Lehmann, 1999) free of nuisance parameters. No pivot exists for the ICC in the two-way layout, but a Generalized Pivotal Quantity (GPQ) does exist. A GPQ is a function of the parameter, the data, and observable values of statistics; like the pivot, the GPQ has a distribution that can be described in terms of known distributions. The GPQ can be used to construct a Generalized Confidence Interval (GCI). The inclusion of observable values means that there is a subtle difference between the definition of the CI and the GCI (Weerahandi, 1993). More discussion appears in the Methods below and in the 1993 reference.

Frequentist methods have been compared in past papers. Cappelleri and Ting (2003) compared the MLS approach in a two-way layout without interaction to a Satterthwaite two moment approach, standard approach, and three moment approach. The MLS was found the best. Gilder et al. (2007) compared MLS and GCI in two-way layouts with an interaction term, and recommended the MLS for overall performance.

GCI APPROACH EQUATIONS

The generalized pivotal quantities are presented in Burdick et al. (2005) and are:

$$
\begin{aligned}
GPQ_{\sigma_b^2} &= \max\left[0, \frac{1}{l_0 r_0}\left\{\frac{SSB}{W_2} - \frac{SSE}{W_3}\right\}\right] \\
GPQ_{\sigma_b^2+\sigma_l^2+\sigma_e^2} &= \frac{SSL}{b_0 r_0 W_1} + \frac{SSB}{l_0 r_0 W_2} + \frac{(BLR - B - L)SSE}{b_0 l_0 r_0 W_3} \\
GPQ_{ICC_b} &= \frac{GPQ_{\sigma_b^2}}{GPQ_{\sigma_b^2+\sigma_l^2+\sigma_e^2}}.
\end{aligned}
$$

Here W_1, W_2 and W_3 are independent chi-squared random variables with degrees of freedom $l_0 - 1$, $b_0 - 1$ and $b_0 l_0 r_0 - b_0 - l_0 - 1$, respectively. The interval is constructed by generating $100,000$ sets of (W_1, W_2, W_3), sorting the resulting GPQ_{ICC_b} from lowest to highest, and taking as the lower limit the $(\alpha/2)*100$th percentile and as upper limit the $(1-\alpha/2)*100$th percentile.

MLS APPROACH EQUATIONS

For completeness, we present the formulas from Cappelleri and Ting (2003). Let S_1^2, S_2^2 and S_3^2 denote the respective mean squares for patients, laboratories and residual errors in a ANOVA table for the two-way random effect model, where $df_i S_i^2/\theta_i \sim \chi^2_{df_i}$ with df_i denoting the degress of freedom. The $100(1-\alpha)\%$ upper confidence limit U for the ICC can be obtained as

$$U = (-B_U + \sqrt{Q_1})/(2A_U)$$

with $Q1 = \max(0, B_U^2 - 4A_U C_U)$, where $A_U = [(1-H_1^2)S_1^4 + (1-G_2^2)d_2^2 S_2^4 + (1-G_3^2)d_3^2 S_3^4 + (2+H_{12})d_2 S_1^2 S_2^2 + (2+H_{13})d_3 S_1^2 S_3^2 + 2d_2 S_2^2 d_3 S_3^2]$, $B_U =$

$[-2(1-H_1^2)S_1^4 + 2(1-G_3^2)d_3 S_3^4 - (2+H_{12})d_2 S_1^2 S_2^2 - (2+H_{13})(d_3-1)S_1^2 S_3^2 + 2d_2 S_2^2 S_3^2]$, $C_U = (1-H_1^2)S_1^4 + (1-G_3^2)S_3^4 - (2+H_{13})S_1^2 S_3^2$ with $d_2 = I/J$, $d_3 = I - 1 - (I/J)$, $H_i = (1/F_{\alpha:df_i,\infty}) - 1$, $G_i = 1 - (1/F_{1-\alpha:df_i,\infty})$, $H_{1j} = [(1-F_{\alpha:df_1,df_j})^2 - (H_1 F_{\alpha:df_1,df_j})^2 - G_j^2]/F_{\alpha:df_1,df_j}$ and $G_{1j} = [(F_{1-\alpha:df_1,df_j} - 1)^2 - (G_1 F_{1-\alpha:df_1,df_j})^2 - H_j^2]/F_{1-\alpha:df_1,df_j}$.

The $100(1-\alpha)\%$ lower confidence limit L can be similarly obtained as

$$L = (-B_L - \sqrt{Q_2})/(2A_L)$$

with $Q2 = \max(0, B_L^2 - 4A_L C_L)$, where A_L, B_L and C_L can be obtained from the expressions of A_U, B_U and C_U by replacing H with G and G with H , respectively.

Finally, an approximate two-sided $100(1-\alpha)\%$ confidence interval can be derived from U and L by replacing α with $\alpha/2$ in the expressions for H_i, G_i, H_{1j} and G_{1j}.

Section S2: A REVIEW OF BAYESIAN METHODS

Bayesian intervals are defined within the Bayesian paradigm. In that paradigm, there is a prior distribution on variance components before the experiment, and a posterior distribution after the experiment that reflects how the observed data modified the prior. A 95% Bayesian (credible) interval contains 95% of the posterior probability, that is, the probability under the posterior density curve. The posterior distribution is proportional to the prior times the likelihood, so that a change in the prior also changes the posterior distribution. When scientists are reporting results, this dependence of a reported interval on the specified prior can distract from the findings; readers may wonder how much the interval depends on the prior used. This leads to non-informative priors which are appropriate "when no prior data exists or when inference based solely on the data is desired" (Carlin and Louis, 2009, p. 36). Non-informative priors have been used

extensively in random effects models and shown to have good frequentist performance in some of these settings (Browne and Draper, 2006).

Due to the wide availability of Bayesian computing software, Bayesian credible intervals for the ICC are increasingly being reported in the biomedical literature (Barzman et al., 2012).

Bayesian approaches to linear models are discussed in Box and Tiao (1992) and Carlin and Louis (2009). Bayesian methods for fitting variance components are reviewed in Browne and Draper (2006). They report frequentist properties of the Bayesian methods for nested designs. In contrast to that work, we examine a crossed, rather than nested, model. Bayesian credible interval methods for parameters in one-way random effect linear models with priors on variance components are reviewed extensively in Gelman (2006). That paper considers a variety of non-informative prior distributions. Gelman showed that the commonly used non-informative inverse gamma priors result in credible intervals for variance components which can perform poorly because they are very sensitive to the choice of the prior parameters. After examining a wide range of alternative distributions, Gelman (2006) recommended improper uniform priors on standard deviations for this one-way ANOVA setting. In a subsequent application, Gelman used a mildly informative prior. Gelman did not in this paper study the crossed, random effects model.

Section S3: INVESTIGATION OF THE BAYESIAN INTERVAL METHODS

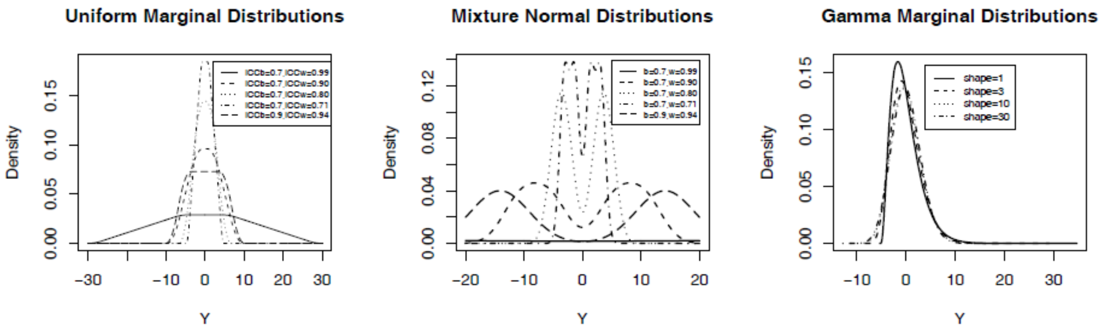

(a) (b) (c)

Figure S1: Marginal distribution of the responses for the non-normal simulations. Uniform (a) and mixture normal (b) densities based on mathematical formulas presented in text. For the (shifted, scaled) gamma (rightmost plot) ICC_b=0.70 and ICC_w=0.90. For the gamma (c), density estimates from 10 million Monte Carlo simulations.

Priors for the ICC_b and ICC_w that are induced by the inverse gamma, IG(0.001,0.001), distribution on each variance component are derived in the supplement and plotted in Figure 1(a). The priors are both nearly

degenerate, with point masses at 0 and 1. As discussed in Carlin and Louis (2009), the noninformative inverse gamma distribution has a very heavy tail. This means that one variance parameter is likely to be much larger than one or both of the others. The ICC_b prior is zero or one when one or more of the three parameters $\sigma_b^2, \sigma_l^2, \sigma_e^2$ is infinite relative to the other(s). For example, on a typical modern personal computer (PC), the largest machine number is about 2×10^{308}. But, for X~IG(0.001,0.001), $P(X>2\times10^{308})\approx0.49$. So, about 49% of the random variables will be larger than the largest number on a typical PC, i.e., effectively infinite. Suppose $\sigma_b^2=\infty$. Then $ICC_b=\infty/(\infty+\sigma_l^2+\sigma_e^2)=1$. On the other hand, if $\sigma_b^2<\infty$, but $\sigma_e^2=\infty$, then $ICC_b=\sigma_b^2/(\sigma_b^2+\sigma_l^2+\infty)=0$. This explains the extreme finding in Figure 1(a). See Supplement Section S7 for more discussion.

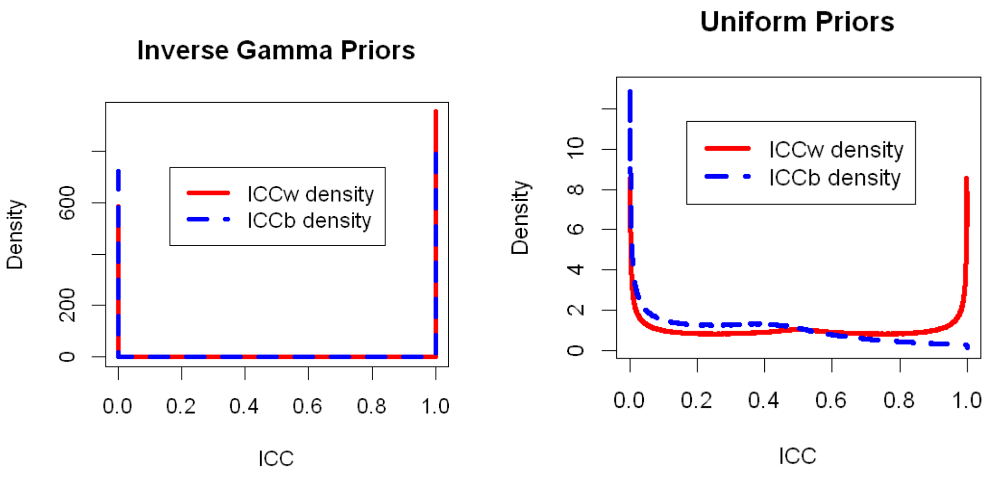

(a) (b)

Figure S2: Prior distributions on the intraclass correlation coefficients, between-lab (ICC_b) and within-lab (ICC_w).

If the priors on each standard deviation component are mutually independent and uniform over the positive real numbers, then the prior distribution of the between-lab intra-class correlation ($ICC_b=\rho_b$) and the within-lab intra-class correlation ($ICC_w=\rho_w$) are derived in Supplement Section S1. The priors are

$$f(\rho_b) = \int_{w=0}^{w=\min(1/\rho_b, 2/(1-\rho_b))} \frac{w^{1/2} k(w(1-\rho_b))}{8\sqrt{\rho_b}} 1_{\{2 > w(1-\rho_b) > 1\}} dw +$$

$$\int_{w=0}^{w=\min(1/\rho_b, 2/(1-\rho_b))} \frac{w^{1/2} k(w(1-\rho_b))}{8\sqrt{\rho_b}} 1_{\{2 > w(1-\rho_b) > 1\}} dw$$

$$f(\rho_w) = \frac{1}{4}(\rho_w)^{-1/2}(1-\rho_w)^{-3/2} 1_{\{\rho_w < 1/2\}} + (\rho_w)^{-3/2}(1-\rho_w)^{-1/2} 1_{\{\rho_w \geq 1/2\}}$$

where $k(x) = \int_{x-1}^{x} u^{-1/2}(1-u)^{-1/2} du$.

These prior densities are plotted in Figure 1b. As can be seen, the ICC_w prior places most of its weight near 0 and 1. But the density is spread out enough to be non-degenerate across the range of possible values. For example, under this prior $P(0.001 < ICC_w < 0.999) > 0.99$. The ICC_b density places more prior weight near zero compared to the ICC_w prior, but is also non-degenerate. For this prior, $P(0.001 < ICC_b < 0.999) > 0.99$.

2.3 Choosing Bayesian priors

We investigated the performance of inference using the classical noninformative inverted gamma distribution. A range of inverse gamma were considered, with focus on the popular IG(0.001,0.001) prior defined by the probability density function $f(x) = Exp[-1/(0.001x)]/\{\Gamma(0.001)0.001^{0.001}x^{1.001}\}$. This is the same as an inverted Wishart distribution. Our research found a number of problems with this prior:

1. When data were generated from the model of Equation (1), the coverage probabilities of these intervals varied depending on true values of the population parameters. But, in real applications, these population parameters are unknown. For example, a 95% credible interval achieved 0.945 coverage when ICC_w=0.80, but this dropped to 0.880 when ICC_w=0.99, where ICC_b=0.70 in both cases. The coverage depends in complicated ways on the underlying truth.

2. The confidence intervals were not invariant under scale transformations (Lehmann, 1986). In other words, when the true data are transformed by a multiplicative factor c>0, resulting in $(\sigma_b^2, \sigma_l^2, \sigma_e^2) \rightarrow (c\sigma_b^2, c\sigma_l^2, c\sigma_e^2)$, then the confidence interval changed as well. The ICC itself is scale invariant, since $ICC_b = c\sigma_b^2 / (c\sigma_b^2 + c\sigma_l^2 + c\sigma_e^2) = \sigma_b^2 / (\sigma_b^2 + \sigma_l^2 + \sigma_e^2)$. For example, the mean width of the confidence interval for ICC_b changed from 0.42 to 0.54 when we changed from c=1 to c=0.01 (with σ_e^2=1, ICC_w=0.9, ICC_b=0.70, based on 10,000 simulations). In practical terms, this means that converting units from parts-per-million to parts-per-ten-thousand, say, would result in much different IG-prior Bayesian intervals.

These two problems remained under a wide variety of noninformative inverse gamma prior parameter settings examined. Therefore, the inverse gamma results are not presented, and only Bayesian results for the uniform distribution on standard deviations are studied. The uniform distribution displayed neither of these problems, has been proposed as a gold standard in similar settings (Gelman, 2006), and can be implemented with existing widely-available software.

DETAILS OF THE INVERSE GAMMA PRIOR

The prior densities when inverse gamma distributions, IG(0.001,0.001), are placed on all three variance components are nearly degenerate. The prior distribution for ICC_w has a Beta distribution that satisfies P(0.001< ICCw <0.999)<0.007. The prior density for ICCw is extremely highly concentrated near 0 and 1 and is close to degenerate. The prior density for ICCb does not have a closed form, but is also close to degenerate in the same way, with high concentration near 0 and 1. To confirm the mathematical derivations shown in the density plots, we ran simulations as well. Based on 1,000,000 simulations, we estimated the probability P(.001<ICCb<0.999)<.003.

Similar problems were encountered when inverse gamma distributions were combined with other popular priors. If the priors on the biological and laboratory variances are folded F(2,2,1), and the prior on the error is IG(0.001,0.001), then P(0.001<ICCb <0.999)<0.001. Similarly, if the priors on biological and laboratory variances are half Cauchy(0,3), and the prior on the error variance is IG(0.001, 0.001), then P(0.001< ICCb <0.999)<0.02. This shows that near degeneracy is created even if only one variance parameter has the IG distribution. This supports the conclusion, as discussed in Carlin and Louis, that the heavy tails of the inverse gamma distribution induce unfavorable Bayesian inference.

SOME CHECKING OF THE BAYESIAN PRIOR DERIVATIONS

To evaluate the equations in the paper, pure Monte Carlo was used to generate large numbers of samples from the derived distribution. In this section appear the density estimates from the pure Monte Carlo together with the density estimates from the equations in the paper, together on the same plot, for the equations of the induced prior distributions for the inverse gamma and uniform distributions.

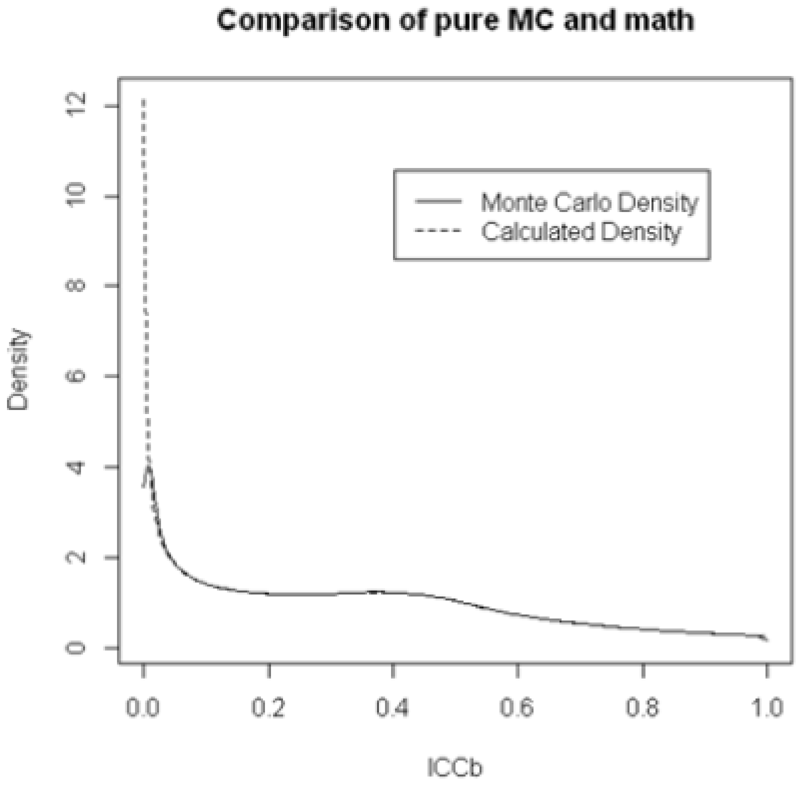

Figure 1: ICCb for the uniform distributions on standard deviations. Calculated density from paper formula and Monte Carlo density estimate based on 10 million Monte Carlo observations.

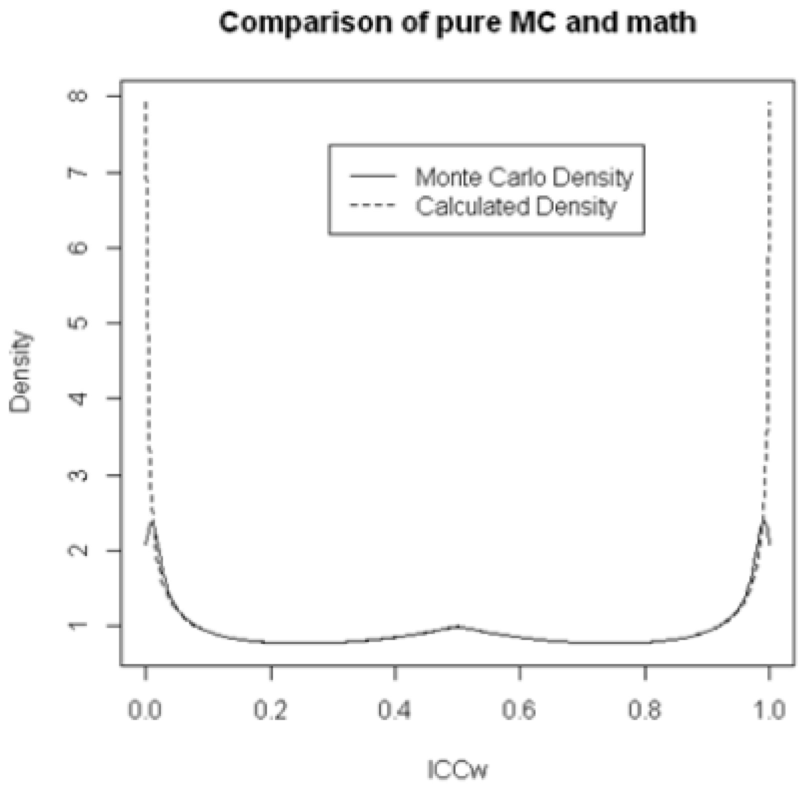

Figure 2: ICCw for the uniform distributions on standard deviations. Calculated density from paper formula and Monte Carlo density estimate based on 10 million Monte Carlo observations.

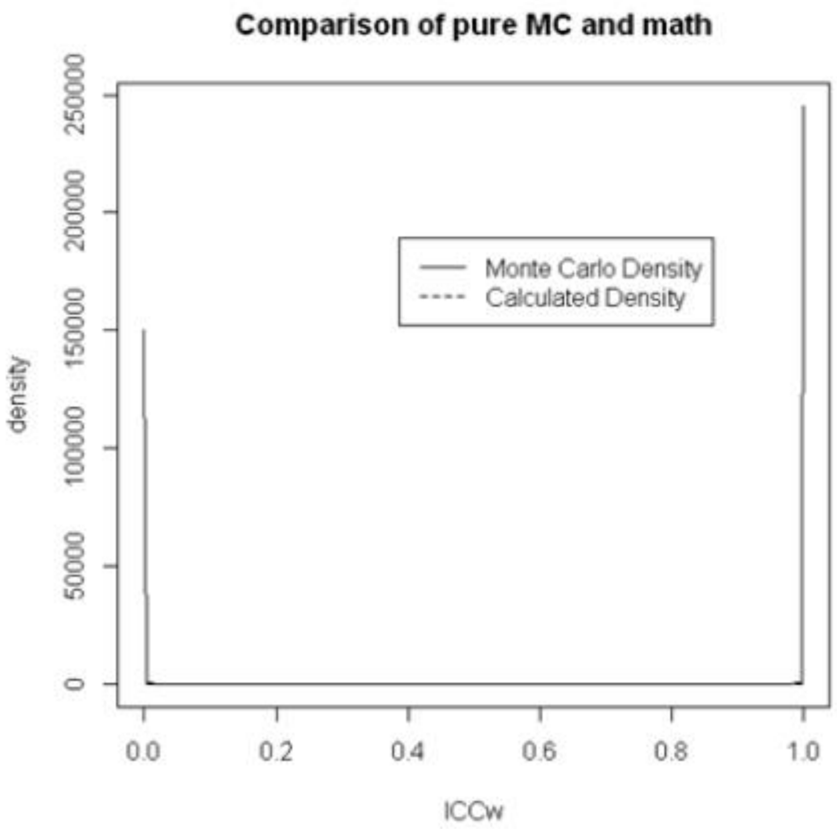

Figure 3: Comparison of math and Monte Carlo for the Inverse gamma density estimation.

Due to numerical integration problems with the distributions, the ICCb for the IG(0.001,0.001) was restricted to Monte Carlo integration.

Section S4: SAMANIEGO CRITERIA

Samaniego (2010) recommends using the Bayes risk of an estimator, relative to the true prior, say G_0, as a criterion for judging the superiority of one estimator over another. (Note that this is in contrast to traditional Bayesian analysis, which considers the loss relative to the "operational prior" – which is the one used in the estimation procedure.) In the context of interval estimation, a common Bayes loss function (e.g., Carlin and Louis, p. 434) for an interval estimate $\hat{a}$ is

$$L(\rho_b, \hat{a}) \quad = \quad I_{\{\rho_b \notin \hat{a}\}} + c \times Volume(\hat{a}).$$

Here, c is a constant controlling the tradeoff between volume and posterior probability of coverage. Viewing the two components on the right separately, one can compute the Bayes risk for each associated with a point-mass prior.

Recall that the Bayes risk with respect to a prior distribution $\rho_b \sim G$ is defined as (Berger, Def. 6, 1985) $r(\rho_b, \hat{a}; G) = E_G[R(\rho_b, \hat{a})]$ where the notation E_F means take the expectation with respect to the distribution F, and R is the risk function, $R(\rho_b, \hat{a}) = E_{D|\mathcal{M}}[L(\rho_b, \hat{a})]$, where $D|\mathcal{M}$ is the distribution of the data given the model, $\mathcal{M}$. If we consider G_0 to be the true prior, represented at a point mass at $\rho_0 \in [0, 1]$, then the first component of the Bayes risk with respect to G_0 is

$$\begin{aligned} r_1(\rho_b, \hat{a}; G_0) &= E_{G_0}\left\{E_{D|\mathcal{M}}[I_{\{\rho_b \notin \hat{a}\}}]\right\} \\ &= Prob(\rho_b \notin \hat{a} | \rho_b = \rho_0). \end{aligned}$$

This is the frequentist probability that ρ_b is not in the interval if, in fact, $\rho_b = \rho_0$. In the tables are given estimates of one minus this quantity, corresponding the coverage probability. The second component of the Bayes risk is

$$\begin{aligned} r_2(\rho_b, \hat{a}; G_0) &= E_{G_0}\left\{E_{D|\mathcal{M}}[c \times Volume(\hat{a})]\right\} \\ &= c \times E_{G_0}[Volume(\hat{a})] \\ &\propto \mu_{volume|\rho_b=\rho_0}. \end{aligned}$$

This component of the risk is proportional to the average interval length when, in fact, $\rho_b = \rho_0$. In the tables are given estimates of the average interval lengths. In sum, the tables provide these two key components for evaluating the Bayes risk with respect to the true prior under various true priors.

Section S5: DESCRIPTION OF THE EXCEL FILES

FILENAME: Summary 201212-update.xls

Design: Index of the simulation settings used

Method: The method used to calculate the confidence interval. GPQ= Generalized confidence interval method; MLSCI = modified large-sample confidence interval method; Unif = Bayesian credible interval using uniform priors on standard deviations.

Center_mean: The average midpoint of the confidence intervals.

Center_sd: The standard deviation of the confidence interval midpoints.

Width_mean: The average width of the confidence intervals.

Width_sd: The standard deviation of the confidence interval widths.

Coverage: The proportion of simulation CIs that contained the true value.

N: Number of biological replicates

Labs: Number of labs

ICCW: Within-lab intraclass correlation coefficient

FILENAME: supp_Summary 20140712-194908.xls presents results based on 10,000 simulations per table row.

- **Design**: Two numbers with first being 100 times the within-lab ICC, and the second being the number of laboratories in the design (denoted l_0). The between-lab ICC is fixed at 0.70. Number of biological replicates is fixed at 24 (denoted b_0).
- **Method:** The confidence interval construction method used, either GCI, MLS or Bayes with uniform prior on standard deviations.
- **Center_mean:** The average of the midpoints of the confidence intervals.
- **Center_sd**: The standard deviation of the midpoints of the confidence intervals.
- **Width_mean:** The average width of the confidence intervals.
- **Width_sd:** The standard deviation of the widths of the confidence intervals.
- **Coverage:** The empirical coverage observed.

- **Coverage>0.945:** Indicator of whether column to the left is greater than 0.945.
- **Abs bias in coverage:** Bias of empirical coverage relative to nominal 95%.
- **Mean bias in coverage – Bayes:** The average bias in the Bayesian coverage across all simulations.

FILENAME: supp_Coverage for Labs 4 to 7.xls presents results based on 10,000 simulations per table row.

- **Design**: Two numbers with first being 100 times the within-lab ICC, and the second being the number of laboratories in the design (denoted l_0). The between-lab ICC is fixed at 0.70. The number of biological replicates is fixed at 24 (denoted b_0).
- **Method:** The confidence interval construction method used, either GCI, MLS or Bayes with uniform prior on standard deviations.
- **Center_mean:** The average of the midpoints of the confidence intervals.
- **Center_sd**: The standard deviation of the midpoints of the confidence intervals.
- **Width_mean:** The average width of the confidence intervals.
- **Width_sd:** The standard deviation of the widths of the confidence intervals.
- **Coverage:** The empirical coverage observed.

FILENAME: supp_ICC Estimates.xls presents results based on 10,000 simulations per table row.

- **Design**: Two numbers with first being 100 times the within-lab ICC, and the second being the number of laboratories in the design (denoted l_0). The between-lab ICC is fixed at 0.70. The number of biological replicates is fixed at 24 (denoted b_0).
- **ICChatMean:** The mean value of the estimated between-lab ICC, using the formula in Saito et al. (2006).

- **ICChatSD:** The standard deviation of the estimated between-lab ICC.
- **Model:** The model used to generate the data; for the gamma, given is the alpha parameter.

Section S6: MARGINAL DISTRIBUTIONS FOR SIMULATION SETTINGS

First, for the uniform simulation setting, to find the marginal distribution of Y_{blr}, we can note that $B_b = U_b - A_b$ where U_b is uniform on $(0, 2A_b)$, and similarly for L_l and e_{blr}. If $W = U_b + U_l + U_e$, then (Buonocore et al., 2009)

$$\begin{aligned} f_w(w) \;\; = \;\; & \Big\{max(x,0)^2 - max(x-2A_b,0)^2 - max(x-2A_l,0)^2 - max(x-2A_e,0)^2 \\ & +max(x-(2A_b+2A_l),0)^2 + max(x-(2A_b+2A_e),0)^2 + \\ & max(x-(2A_l+2A_e),0)^2 \\ & -max(x-(2A_b+2A_l+2A_e),0)^2\Big\} / (2 * 2A_b * 2A_l * 2A_e). \end{aligned}$$

Finally, noting that $f(y_{blr}) = f_w(y_{blr} + A_b + A_l + A_e)$ gives the marginal density function of the response.

Now, for the mixture normal simulation settings, let $\phi(\cdot; \mu, \sigma^2)$ be the normal density with mean μ and variance σ^2. Then $Y_{blr} = B_b + L_l + e_{bl}$

where $B_b = 0.5\phi(\cdot;\mu_b,\mu_b^2/9) + 0.5\phi(\cdot;-\mu_b,\mu_b^2/9)$, $L_l = 0.5\phi(\cdot;\mu_l,\mu_l^2/9) + 0.5\phi(\cdot;-\mu_l,\mu_l^2/9)$ and $e_{bl} = 0.5\phi(\cdot;\mu_e,\mu_e^2/9) + 0.5\phi(\cdot;-\mu_e,\mu_e^2/9)$. The resulting density is a mixture of 8 normal random variables with probability 0.125 on each.

For the mixture gamma simulation settings, a weighted sum of gammas does not have a closed form density function. Therefore, we estimated the density by Monte Carlo.

Section S7: TABLE OF SIMULATION SETTINGS

Supplement Table S1: Simulation parameter settings: Gammas scaled to have variances identical to corresponding normal simulation.

Distributions	ICC_b	ICC_w	Parameter settings
Normal	0.70	0.99	$\sigma_e^2 = 1, \sigma_l^2 = 41.43, \sigma_b^2 = 99$
Normal	0.70	0.90	$\sigma_e^2 = 1, \sigma_l^2 = 9, \sigma_b^2 = 2.86$
Normal	0.70	0.80	$\sigma_e^2 = 1, \sigma_l^2 = 4, \sigma_b^2 = 0.71$
Normal	0.70	0.71	$\sigma_e^2 = 1, \sigma_l^2 = 2.45, \sigma_b^2 = 0.05$
Normal	0.90	0.94	$\sigma_e^2 = 1, \sigma_l^2 = 15.67, \sigma_b^2 = 0.74$
Gamma	0.70	0.99	$\beta \equiv 1, \alpha = 1, 3, 10, 40$
Gamma	0.70	0.90	$\beta \equiv 1, \alpha = 1, 3, 10, 40$
Gamma	0.70	0.80	$\beta \equiv 1, \alpha = 1, 3, 10, 40$
Gamma	0.70	0.71	$\beta \equiv 1, \alpha = 1, 3, 10, 40$
Gamma	0.90	0.94	$\beta \equiv 1, \alpha = 1 = 1, 3, 10, 40$
Mixture Normal	0.70	0.99	$\mu_e^2 = 0.9, \mu_l^2 = 89.1, \mu_b^2 = 37.287$
Mixture Normal	0.70	0.90	$\mu_e^2 = 0.9, \mu_l^2 = 2.574, \mu_b^2 = 8.1$
Mixture Normal	0.70	0.80	$\mu_e^2 = 0.9, \mu_l^2 = 0.639, \mu_b^2 = 3.6$
Mixture Normal	0.70	0.71	$\mu_e^2 = 0.9, \mu_l^2 = 0.045, \mu_b^2 = 2.205$
Mixture Normal	0.90	0.94	$\mu_e^2 = 0.9, \mu_l^2 = 1.216, \mu_b^2 = 14.103$
Uniform	0.70	0.99	$A_e = \sqrt{3}, A_l = \sqrt{297}, A_b = \sqrt{124.29}$
Uniform	0.70	0.90	$A_e = \sqrt{3}, A_l = \sqrt{8.58}, A_b = \sqrt{27}$
Uniform	0.70	0.80	$A_e = \sqrt{3}, A_l = \sqrt{2.13}, A_b = \sqrt{12}$
Uniform	0.70	0.71	$A_e = \sqrt{3}, A_l = \sqrt{0.15}, A_b = \sqrt{7.35}$
Uniform	0.90	0.94	$A_e = \sqrt{3}, A_l = \sqrt{2.22}, A_b = \sqrt{47.01}$

Section S8: SUPPLEMENTARY FIGURES

Mixture normal effects and errors

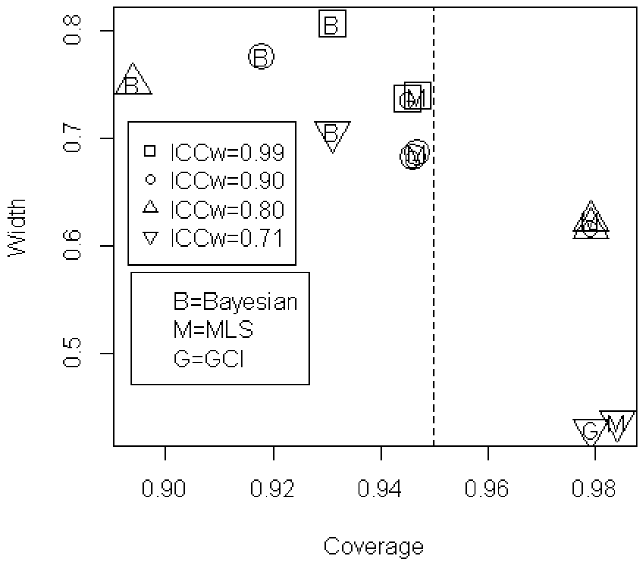

Figure above: Simulation study with 48 biological replicates and 3 labs for a total of 144 observations. Comparison of MLS, GPQ and Bayes method performance. Nominal 95% confidence intervals. Coverages and average widths calculated from 10,000 simulations. (a) Mixture normal model data. 48 biological replicates and 3 labs for a total of 144 observations.

Section S9: DATA ANALYSIS DETAILS

Raw data CEL files and covariate sample data were downloaded from the National Cancer Institute's Center for Bioinformatics' (NCICB) caArray 2.0 website (https://array.nci.nih.gov). The Experiment Identifier is dobbi00100. Data on the tumor samples were normalized using the Bioconductor affy suite (Gautier et al., 2004). The package's mas5 function was applied under R version 2.15.2. Further normalization consisted of identifying the subset of features with mas5 present calls for over half the arrays, then multiplying each array value by a constant to make the median of this feature set 500. Tumors with signal values below 5.0 were truncated to 5.0, and the base 2 logarithm transformation applied.

REFERENCES

Barzman, D., Mossman, D., Sonnier, L., and Sorter, M. (2012). Brief rating of aggression by children and adolescents (BRACHA): A reliability study. *Journal of the American Academy of Psychiatry and the Law* **40**, 374-382.

Box GEP and Tiao GC (1973) *Bayesian Inference in Statistical Analysis*. Wiley, New York.

Browne WJ and Draper D (2006) A comparison of Bayesian and likelihood-based methods for fitting multilevel models. *Bayesian Analysis*, **3,** 473-514.

Cappelleri JC and Ting N (2003) A modified large-sample approach to approximate interval estimation for a particular intraclass correlation coefficient. *Statistics in Medicine*, **22**, 1861-1877.

Carlin BP and Louis TA (2008) *Bayesian Methods for Data Analysis, Third Edition*. Chapman and Hall, Boca Raton.

Gelman, A. (2006). Prior distributions for variance parameters in hierarchical models. *Bayesian Analysis* **1(3)**, 515-533.

Gautier L, Cope L, Bolstad BM, Irizarry RA (2004) Affy – analysis of Affymetrix GeneChip data at the probe level. *Bioinformatics*, **20**, 307-315.

Gilder K, Ting N, Tian L., Cappelleri JC, Hanumara RC (2007) Confidence intervals on intraclass correlation coefficients in a balanced two-factor random design. *Journal of Statistical Planning and Inference*, **137**, 1199-1212.

Graybill FA and Wang C (1980) Confidence intervals on nonnegative linear combinations of variances. *Journal of the American Statistical Association.* 75: 869-873.

Lehmann EL (1986) *Testing Statistical Hypotheses, Second Edition*. Springer-Verlag, New York.

Lehmann EL and Casella G (1998) *Theory of Point Estimation.* Springer-Verlag, New York.

Lehmann EL (1999) *Elements of Large-Sample Theory*. Springer-Verlag, New York.

Searle SR, Casella G, McCulloch CE (2006) *Variance Components*. Wiley, New York.

Weerahandi S (1993) Generalized confidence intervals. *Journal of the American Statistical Association*, **88**, 899-905.

Zou KH and McDermott MP (1999) Higher-moment approaches to approximate interval estimation for a certain intraclass correlation coefficient. *Statistics in Medicine*, **18,** 2051-2061.
